# Analysis of remaining motion using one innovative upper airway opening cervical collar and two traditional cervical collars

**DOI:** 10.1038/s41598-021-00194-w

**Published:** 2021-10-18

**Authors:** Matthias K. Jung, Gregor V. R. von Ehrlich-Treuenstätt, Holger Keil, Paul A. Grützner, Niko R. E. Schneider, Michael Kreinest

**Affiliations:** 1 BG Trauma Center Ludwigshafen, Clinic for Trauma and Orthopaedic Surgery, University of Heidelberg, Ludwig-Guttmann-Str. 13, 67071 Ludwigshafen on the Rhine, Germany; 2 Clinic for Trauma and Orthopaedic Surgery, Universitätsklinikum Erlangen, Krankenhausstraße 12, 91054 Erlangen, Germany; 3 Clinic of Anesthesiology, University of Heidelberg, Im Neuenheimer Feld 420, 69120 Heidelberg, Germany

**Keywords:** Preclinical research, Musculoskeletal system, Trauma

## Abstract

The aim of this study was to compare the remaining motion of an immobilized cervical spine using an innovative cervical collar as well as two traditional cervical collars. The study was performed on eight fresh human cadavers. The cervical spine was immobilized with one innovative (Lubo Airway Collar) and two traditional cervical collars (Stifneck and Perfit ACE). The flexion and lateral bending of the cervical spine were measured using a wireless motion tracker (Xsens). With the Weinman Lubo Airway Collar attached, the mean remaining flexion was 20.0 ± 9.0°. The mean remaining flexion was lowest with the Laerdal Stifneck (13.1 ± 6.6°) or Ambu Perfit ACE (10.8 ± 5.8°) applied. Compared to that of the innovative Weinmann Lubo Airway Collar, the remaining cervical spine flexion was significantly decreased with the Ambu Perfit ACE. There was no significant difference in lateral bending between the three examined collars. The most effective immobilization of the cervical spine was achieved when traditional cervical collars were implemented. However, all tested cervical collars showed remaining motion of the cervical spine. Thus, alternative immobilization techniques should be considered.

## Introduction

Although evidence of the effectiveness of cervical collars is rare^1–3^, the use of rigid cervical collars in preclinical trauma care is known to be a common immobilization technique. The importance of immobilization of the cervical spine is recognized in national and international recommendations for initial trauma care. The application of the cervical collar to avoid secondary injuries of the cervical spine^4,5^ is recommended as one of the first emergency measures^6,7^.

Nevertheless, immobilization of the cervical spine by rigid collars is controversially discussed in the literature^8–12^. Cervical spine immobilization with collars can be accompanied by disadvantages, such as pain, discomfort^13,14^ or pressure ulceration^13,15,16^. Furthermore, the application of a cervical collar can also lead to considerably serious and even life-threatening complications. In particular, restricted respiratory function^17^ and difficult airway management^18^ have been described, which can quickly lead to severe complications or even death, especially in trauma patients^7^. To ease airway management, an innovative cervical collar has been developed that should combine cervical spine immobilization and airway protection. This innovative cervical collar is equipped with a flexible belt for the mandible^19^. While this innovative collar seems to succeed in airway protection, the quality of cervical spine immobilization has not been proven, so far. However, the effectiveness of a cervical collar depends on its perfect fit^20–22^.

The aim of this study was to compare the remaining motion of the cervical spine using an innovative cervical collar as well as two traditional cervical collars on fresh human cadavers.

## Materials and methods

### Study design

The study was performed on fresh human cadavers. The body donors were given detailed information before death and had to give their written informed consent to the body donation. After death, the body was made available for scientific research purposes. Fresh human cadavers were briefly frozen after death. For the experiments, the bodies were thawed to room temperature. This method enabled the simulation of the joint elasticity and soft tissue situation of a living body. Current biomechanical studies do not describe any significant difference in cervical spine motion between fresh human cadavers and patients^4,23,24^.

The inclusion criteria for the current study were as follows: (1) existing written informed consent for body donation for scientific research; (2) no injuries, diseases or operations on the cervical spine; and (3) complete medical records. The complete medical history and the existing medical data of all examined patients were analyzed. Patients with diseases such as tumors, thyroid diseases or similar conditions were excluded from the study.

The local ethics committee reviewed and approved the present study (Ethics Committee, Mainz, Rhineland-Palatinate, Germany, ID: 837.156.16). The study was registered in the German Clinical Trials Register (ID: DRKS00010499). All experiments were performed in accordance with the relevant guidelines and regulations.

### Cervical collars

**Figure 1:**
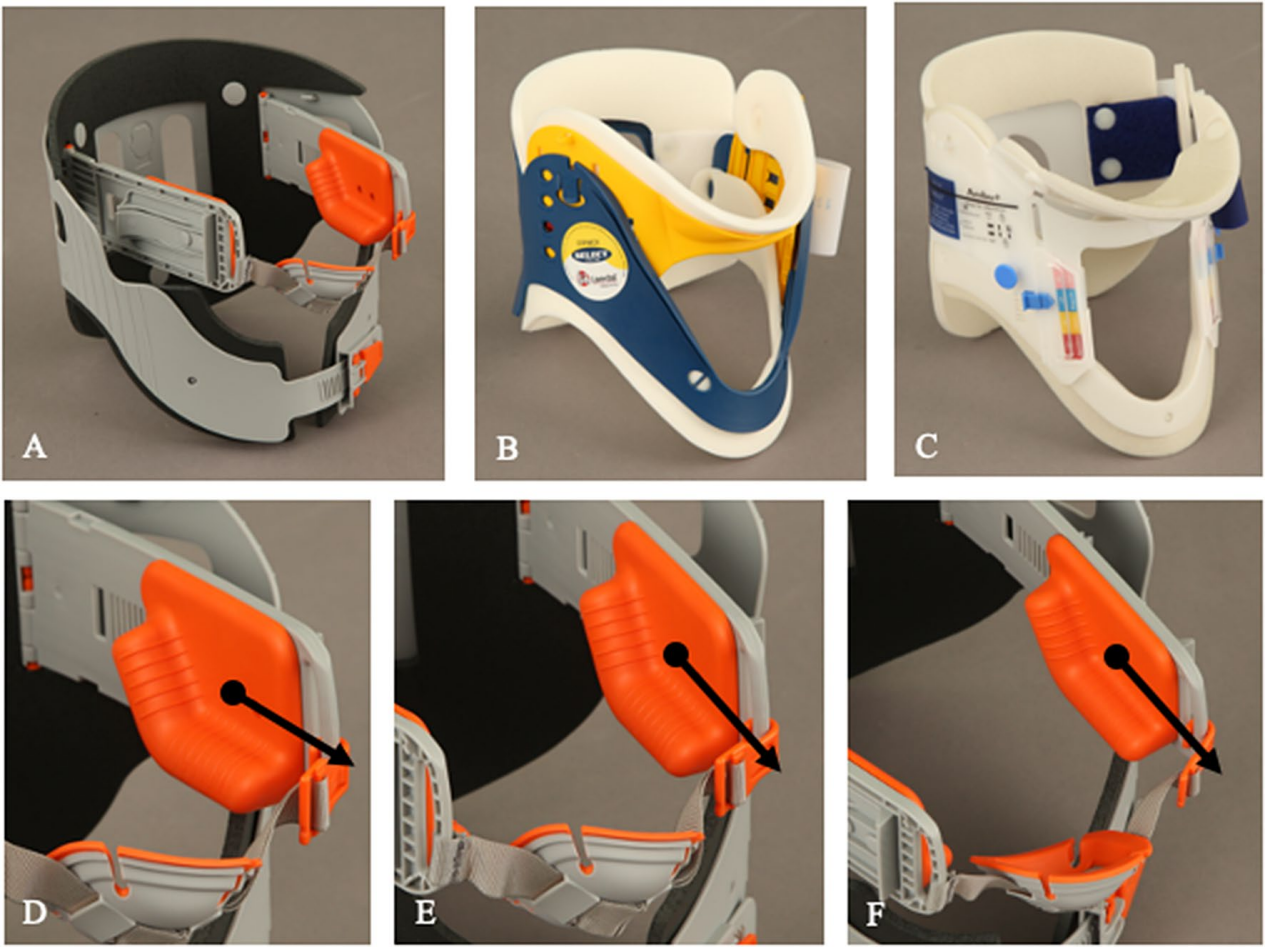
In the present study, three different cervical collars were tested on fresh human cadavers: Weinmann Lubo Airway Collar (**A**), Laerdal Stifneck (**B**) and Ambu Perfit ACE (**C**). Shown is the innovative mechanism (**D**,**E**,**F**). The red angle (black dot) pushes the mandible ventrally (black arrow) for opening the upper airway.

In the present study, three different cervical collars were tested on fresh human cadavers. First, the innovative Weinmann Lubo Airway Collar (Weinmann Emergency Medical Technology GmBh and Co. KG, Hamburg, Germany, Fig. 1A) was tested. This collar was newly developed in 2007 to stabilize the cervical spine and to avoid supraglottic airway obstruction at the same time (Fig. 1D–F) ^19^. Second, two traditional collars were tested, namely, the Laerdal Stifneck (Laerdal Medical GmbH, Puchheim, Germany, Fig. 1B) and the Ambu Perfit ACE (Ambu GmbH, Bad Nauheim, Germany, Fig. 1C). These traditional rigid collars with one-belt fixation have been in use nearly worldwide for many decades.

All cervical collars were adjusted according to the manufacturers' instructions. All different sizes of cervical collars were available during the study. The three cervical collars (Fig. 1A–C) were applied by one experienced emergency medical service (EMS) person to fresh human cadavers. Likewise, the repetitive measurements were performed only by one person experienced in rescue medicine.

### Cervical spine motion measurement

In the present study, the remaining flexion and the remaining lateral bending of the immobilized cervical spine were measured with a wireless human motion tracker system (Xsens Technologies, Enschede, Netherlands). Compared to other methods, this measurement method with a motion tracker has been proven^25–27^ and guarantees exact measurement results^28^. The endpoints of the measurements were maximal flexion and maximal lateral bending.

**Figure 2:**
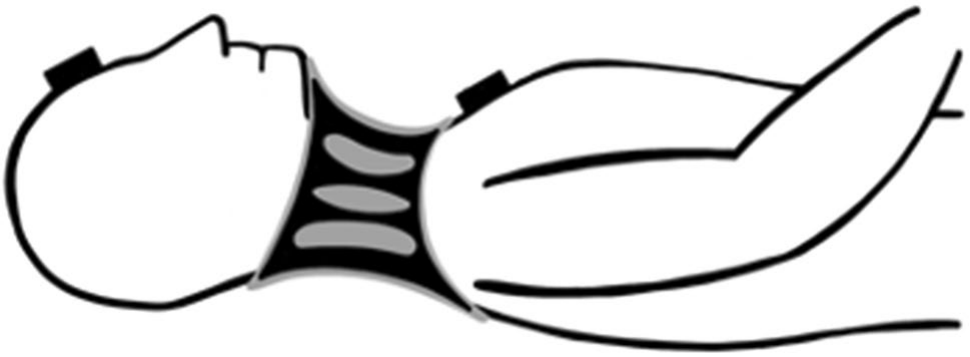
A fresh human cadaver is positioned supine on a spine board with a cervical collar; the motion trackers are fixed to the forehead and to the sternum.

In the standardized motion protocol, the rotation was not included separately since combination motions always occur here. The extension was also not recorded since the fresh human cadavers were lying in the supine position and thus no extension could be measured (Fig. 2).

**Figure 3:**
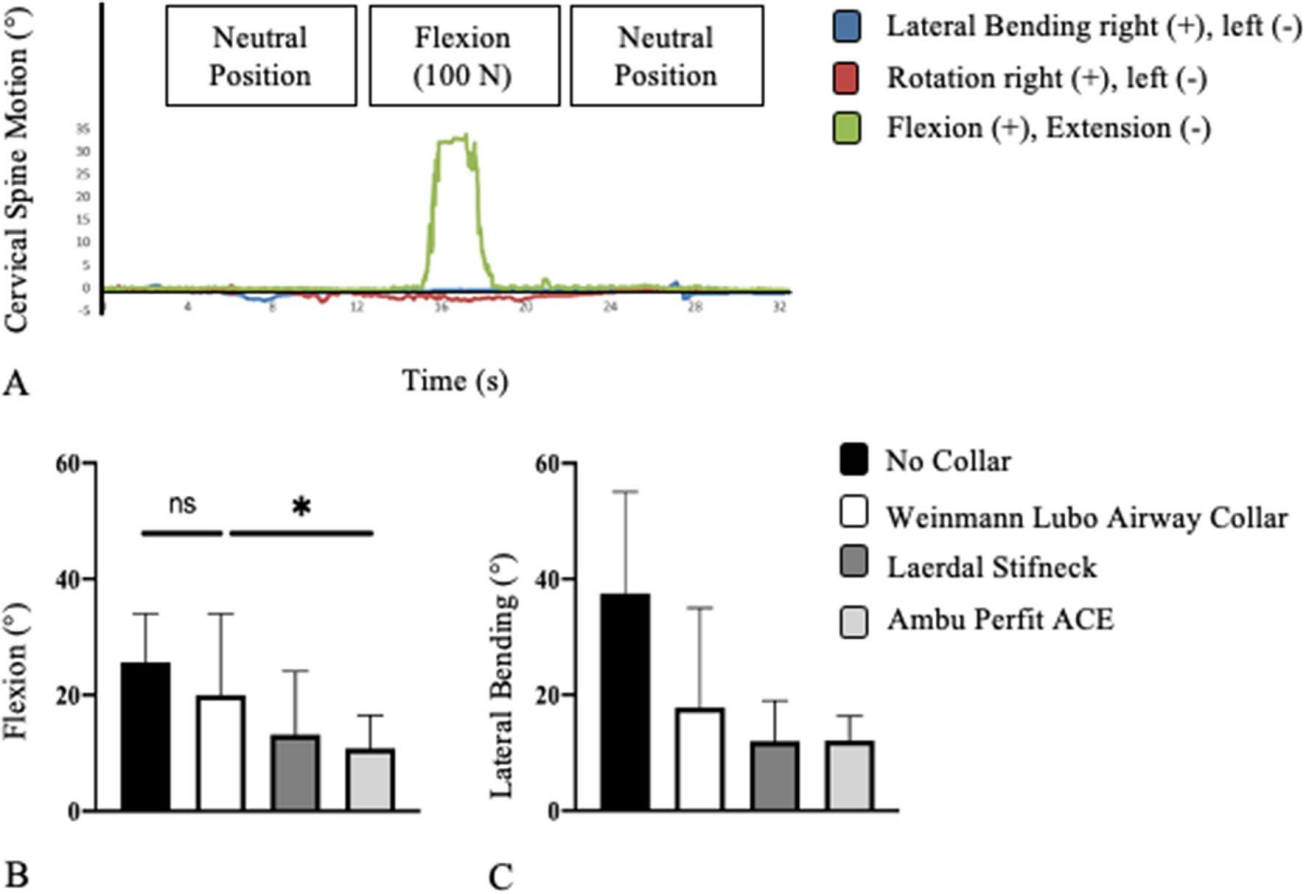
Remaining flexion of the cervical spine if an innovative cervical collar is applied (**A**). Range of remaining flexion of the cervical spine (**B**) and range of remaining lateral bending (**C**) of the three tested cervical collars (**B**,**C**: Only significant differences are marked).

In the experimental setup, fresh human cadavers were placed on a spineboard (Laerdal BaXstrap, Stavanger, Norway) in supine position and fixed by a harness fixation system (MIH-Medical Spiderstrap, Georgsmarienhütte, Germany). One motion tracker was attached to the forehead, and one motion tracker was attached to the thorax of the fresh human cadaver (Fig. 2). The 3D data were synchronized every 20 microseconds by the Xsens recording tool. However, with the help of the motion trackers, rotation and extension were recorded in the respective measured direction of motion (Fig. 3A).

The head of the fresh human cadaver was passively moved ventrally for flexion (Fig. 3A) and to the right for lateral bending. The traction force was 100 N. This force is in the range and in the direction as it may be applied during intubation or patient transport and may therefore initiate or aggravate an assumed spinal injury^3,29,30^. The force was measured with an electronic spring balance (LENI, Fa. Korona, Sundern, Germany).

In the standardized motion protocol, flexion and lateral bending were first analyzed for each fresh human cadaver without the cervical collar (control group). Subsequently, the motion analysis was performed with the three cervical collars in place.

### Statistical data analysis

In the present study, statistical calculations were performed to determine possible differences between the three tested cervical collars. With the D'Agostino and Pearson test, the three groups were tested for normal distribution. The statistical comparison between the four groups was performed using analysis of variance (ANOVA). A *p*-value of < 0.05 was considered statistically significant.

An exploratory analysis of the measured values was performed. For all measurements, descriptive data with the mean, standard deviation and ranges are given. The statistical data analysis was performed with GraphPad PRISM software (Version 8.2.1, San Diego, California, USA).

## Results

### Study collective

Eight fresh human cadavers were included in the present study. The mean age of the cadavers at death was 79.0 ± 12.8 years (range: 56–94 years). The study included five female and three male fresh human cadavers.

### Motion measurement of the cervical spine

The highest remaining flexion of the cervical spine with a cervical collar applied was 34.0° (Fig. 3A). In this case, Weinman Lubo Airway Collar (Fig. 1A) was attached. Overall, the fresh human cadavers with the Weinman Lubo Airway Collar in place had a mean flexion of 20.0 ± 9.0° (range: 10.0–34.0°; Fig. 3B; Table 1). A lower remaining flexion at the cervical spine could be achieved using the two traditional cervical collars (Fig. 1B,C). With the Laerdal Stifneck applied, the mean flexion was 13.1 ± 6.6° (range: 8.0–24.0°). The mean flexion was lowest with the Ambu Perfit ACE (10.8 ± 5.8°; range: 5.0–19.0°; Fig. 3B). Without cervical collar, the fresh human cadavers had a mean cervical flexion of 25 ± 5.0° (range: 18.0°–34.0°, Fig. 3B).

**Table 1:** Results of the remaining motion examination of the three cervical collars and the control group.

	No collar	Weinmann Lubo airway collar	Laerdal Stifneck	Ambu Perfit ACE
Flexion, ° (range)	25.6 ± 5.0 (18.0–34.0)	20.0 ± 9.0 (10.0–34.0)	13.1 ± 6.6 (8.0–24.0)	10.8 ± 5.8 (5.0–19.0)
Lateral bending, ° (range)	37.5 ± 8.7 (29.0–55.0)	17.8 ± 10.2 (7.0–35.0)	12.0 ± 4.8 (7.0–19.0)	12.1 ± 4.3 (6.0–17.0)

The mean flexion of the cervical spine with the Weinmann Lubo Airway Collar on was almost twice as high as that with the Ambu Perfit ACE applied. Compared to the Weinmann Lubo Airway Collar, the remaining cervical spine flexion was significantly (*p* = 0.0474) decreased with the Ambu Perfit ACE (Fig. 3B). The differences in remaining cervical spine flexion between the Laerdal Stifneck and the Weinmann Lubo Airway Collar (*p* = 0.1655) and between the Laerdal Stifneck and the Ambu Perfit ACE (*p* = 0.7923) were not significant. There was no significant difference in cervical flexion with Weinmann Lubo Airway Collar applied or no collar applied (*p* = 0.3611). Between the control examination and the Laerdal Stiffneck (*p* = 0.0049) and the Ambu Perfit ACE (*p* = 0.0008) there was a significant reduction in remaining cervical spine flexion.

The mean remaining lateral bending of the Weinmann Lubo Airway Collar was 17.8 ± 10.2° (range: 7.0–35.0°; Table 1). With the Laerdal Stifneck attached, the mean remaining lateral bending was 12.0 ± 4.8° (range: 7.0–19.0°). The Ambu Perfit ACE allowed for a mean remaining lateral bending of 12.1 ± 4.3° (range: 6.0–17.0°; Figs. 1C and 3C). Without cervical collar, the lateral bending of the cervical spine was 37.5 ± 8.7° (range: 29.0°–55.0°, Fig. 3C).

There was no significant difference in lateral bending between the three examined collars (Weinmann Lubo Airway Collar vs. Ambu Perfit ACE: *p* = 0.2600; Weinmann Lubo Airway Collar vs. Laerdal Stifneck: *p* = 0.2458; Laerdal Stifneck vs. Ambu Perfit ACE: *p* = 0.9993; Fig. 3C). Lateral bending of the cervical spine was significantly reduced by all three cervical collars (*p* < 0.0001).

## Discussion

The aim of this study was to measure and to evaluate quantitatively the remaining motion of the cervical spine during immobilization using one innovative upper airway opening cervical collar and two traditional cervical collars. The present study showed that the attached innovative and traditional cervical collars resulted in significantly different remaining flexion of the immobilized cervical spine. The design of the cervical collars seems to have an influence on their fit and thus on the effectiveness of the immobilization.

The data of the present study showed that compared to traditional cervical collars, the innovative cervical collar allows for twofold increased flexion of the cervical spine. There was no significant difference in the cervical spine flexion whether the innovative cervical collar was applied or not. The novel upper airway opening design is probably decisive for the poor result. The innovative cervical collar has a one-belt system; whereby the mandible is addressed with an extra flexible belt (Fig. 1A). In contrast, both traditional cervical collars address the mandible as well as the occiput, sternum, clavicle, shoulders and upper back with their rigid form (Fig. 1B,C)^19,21,31^. The novel fixation system therefore seems to perform worse than the classic cervical collars in the important flexion motion. The Weinmann Lubo Airway Collar can support airway clearance, but a classic cervical collar should be used in unconscious and intubated patients with suspected cervical spine injury.

According to a study by Lubovsky et al., the innovative Weinmann Lubo Airway Collar, which combines an external airway protector in combination with a cervical collar, appears to be safe and effective in opening and maintaining open airways in anesthetized supine patients^19^. In addition, the authors recommend that further clinical trials are indicated to evaluate the safety and utility of this device under preclinical conditions^19^. To our knowledge, the efficacy of the collar toward cervical spine immobilization has not been tested so far. According to the data of the current study, efficiency of the innovative cervical collar by the means of cervical spine immobilization is worse compared to traditional cervical collars.

The study by Karason et al. also compared widely used cervical collars^32^. The endpoints of the remaining motion were analyzed using a gonimeter. In addition, the intracranial pressure was extrapolated, and the wearing comfort was evaluated subjectively. As in the present study, the classic rigid cervical collars were shown to achieve safe immobilization. Karason et al. were able to determine a remaining motion of immobilization of the cervical spine of 18.0° ± 7.0°. The present study showed significantly better immobilization values. The extent to which this depends on the measurement method used or is even due to the test subjects should be analyzed in further studies.

The motion values that have been measured for the traditional cervical collars were in the same range as in other studies. Hostler et al. found a mean flexion of 9.2° ± 5.0° with the Ambu Perfit ACE applied^33^. Thus, complete immobilization of the cervical spine could not be achieved by a cervical collar alone. These findings are supported by various other studies^3,34^. In addition, many cervical collars are not applied correctly^35^; therefore, their effectiveness is further reduced.

Since a secondary injury or a worsening of an existing injury cannot be completely excluded in cases of remaining cervical spine motion^4,36^, alternative immobilization techniques should be considered. Cervical spine immobilization by a collar could be substantially improved by additionally fixing the patient to a spine board^34^. However, in this case, the main immobilization will derive from fixation on the spineboard, and the cervical collar is dispensable^37,38^. The full body immobilization technique is especially recommended during patient transport^25^.

### Limitations

The present study is limited to some extent. Only human cadavers with an intact cervical spine were analyzed. For patients with an already injured cervical spine, different results can certainly be expected. Furthermore, the cervical spine force was applied in a standardized manner only for flexion and lateral bending. Combined movements were not performed. There is no literature that provides any indication of the magnitude and direction of the force required to cause or even to aggravate a cervical spine injury. Previous studies have shown that after an initial trauma, no further injury or aggravation can occur through further manipulation^39^. However, recent biomechanical studies have shown a direct correlation between manipulation of the severely injured cervical spine and the width of the dural sac^29,30,40^. Furthermore, the application of a cervical collar itself may aggravate a spinal injury^29^. Thus, in future studies, not only the remaining cervical spine motion but also the manipulation that is caused by the collar’s application should be analyzed. Here, significant differences have been previously shown^41^. In addition, it should always be remembered that the cervical support immobilizes best when other immobilization tools are applied^26^. Three cervical collars were examined in the current study; and, according to the literature, many additional cervical collars are available^33^. This efficiency of these other collars should be addressed in further studies.

## Conclusions

The results of the present study indicate that the best immobilization of the cervical spine could be achieved if the cervical collar has a nonmobile, rigid design that is closely attached to the mandible, occiput, sternum, clavicle, shoulders and upper back. However, all tested cervical collars showed remaining motion of the cervical spine. Thus, alternative immobilization techniques should be considered.
